# Enhancing skeletal stability and Class III correction through active orthodontist engagement in virtual surgical planning: A voxel-based 3-dimensional analysis

**DOI:** 10.1016/j.ajodo.2023.09.016

**Published:** 2023-11-27

**Authors:** Selene Barone, Lucia Cevidanes, Felicia Miranda, Marcela Lima Gurgel, Luc Anchling, Nathan Hutin, Jonas Bianchi, Joao Roberto Goncalves, Amerigo Giudice

**Affiliations:** a Department of Health Sciences, School of Dentistry, Magna Graecia University of Catanzaro, Catanzaro, Italy.; b Department of Orthodontics and Pediatric Dentistry, School of Dentistry, University of Michigan, Ann Arbor, Mich.; c Department of Orthodontics, Bauru Dental School, University of São Paulo, Bauru, São Paulo, Brazil.; d Chemistry and Chemical Engineering School - Digital Sciences School Lyon, Lyon, France.; e Department of Orthodontics, Arthur A. Dugoni School of Dentistry, University of the Pacific, San Francisco, Calif.; f Department of Pediatric Dentistry, School of Dentist, São Paulo State University, Araraquara, São Paulo, Brazil.

## Abstract

**Introduction:**

Skeletal stability after bimaxillary surgical correction of Class III malocclusion was investigated through a qualitative and quantitative analysis of the maxilla and the distal and proximal mandibular segments using a 3-dimensional voxel-based superimposition among virtual surgical predictions performed by the orthodontist in close communication with the maxillofacial surgeon and 12-18 months postoperative outcomes.

**Methods:**

A comprehensive secondary data analysis was conducted on deidentified preoperative (1 month before surgery [T1]) and 12-18 months postoperative (midterm [T2]) cone-beam computed tomography scans, along with virtual surgical planning (VSP) data obtained by Dolphin Imaging software. The sample for the study consisted of 17 patients (mean age, 24.8 ± 3.5 years). Using 3D Slicer software, automated tools based on deep-learning approaches were used for cone-beam computed tomography orientation, registration, bone segmentation, and landmark identification. Colormaps were generated for qualitative analysis, whereas linear and angular differences between the planned (T1-VSP) and observed (T1-T2) outcomes were calculated for quantitative assessments. Statistical analysis was conducted with a significance level of α = 0.05.

**Results:**

The midterm surgical outcomes revealed a slight but significantly less maxillary advancement compared with the planned position (mean difference, 1.84 ± 1.50 mm; *P* = 0.004). The repositioning of the mandibular distal segment was stable, with insignificant differences in linear (T1-VSP, 1.01 ± 3.66 mm; T1-T2, 0.32 ± 4.17 mm) and angular (T1-VSP, 1.53° ± 1.60°; T1-T2, 1.54° ± 1.50°) displacements (*P* >0.05). The proximal segments exhibited lateral displacement within 1.5° for both the mandibular right and left ramus at T1-VSP and T1-T2 (*P* >0.05).

**Conclusions:**

The analysis of fully digital planned and surgically repositioned maxilla and mandible revealed excellent precision. In the midterm surgical outcomes of maxillary advancement, a minor deviation from the planned anterior movement was observed.

The primary goal of orthognathic surgery is to correct severe dentofacial deformities, achieving both esthetic and functional improvements.^1^ Traditionally, changes resulting from orthognathic surgery have been analyzed by comparing presurgical and postsurgical 2-dimensional (2D) radiologic images, focusing on skeletal and soft-tissue changes.^2,3^ However, advancements in computer-assisted imaging analysis have made low-dose cone-beam computed tomography (CBCT) available, allowing for 3-dimensional (3D) assessment of orthognathic patients.^4^ Compared with 2D images, CBCT provides more detailed information for surgeons, enabling accurate evaluations of both skeletal and soft tissue and reducing the risk of intraoperative errors.^1,5,6^ The advent of 3D virtual surgical planning (VSP) enhanced preoperative analysis and eliminated many of the laboratory steps involved in surgical splint preparation.^7,8^ Accurate VSP represents a remarkable challenge for orthodontists and orthognathic surgeons, as the virtual plan correction requires collaborative interdisciplinary communication, anticipation of surgical difficulties and limitations, and visual evaluation of the simulated results on both skeletal and soft tissues.^7,9^ The success of orthognathic surgery relies not only on surgical procedures but also on a precise diagnosis and a well-defined treatment plan, aiming for predetermined results, skeletal stability, and long-term soft-tissue harmony during follow-up.^10-17^

For patients with Class III malocclusion, previous studies have observed changes in the mandibular condyles and rami 1 week postsurgery, as well as significant postoperative adaptations 1-year postsurgery.^18,19^ Given that VSP represents a substantial improvement in orthognathic surgical management, it is crucial to analyze the reliability of this protocol by evaluating postoperative outcomes.^1^ Although several studies have reported the accuracy of VSP in the early postoperative months, there is a paucity of data regarding midterm postoperative displacement and remodeling of the jaws after virtually planned orthognathic surgery using an automated voxel-based workflow for 3D imaging analysis.^20-23^ By employing specific mathematical algorithms, a more recent method known as voxel-based registration ensures a high level of accuracy in automated image registration.^2,24^ A comprehensive 3D evaluation of orthognathic patients using a fully digital virtual planning protocol can provide valuable insights into postsurgical displacements of the jaws, quantification of changes, and assessment of their direction. Recognizing the potential for postsurgical skeletal changes and the influence of postsurgical orthodontic movements on skeletal stability, active orthodontist involvement in the virtual planning process is crucial to ensure accurate and stable outcomes.^25^

The objective of this study was to evaluate postsurgical skeletal stability after 12-18 months of follow-up in patients with severe dentoskeletal Class III malocclusions treated with bimaxillary orthognathic surgery after VSP performed by a trained orthodontist in close communication with the oral and maxillofacial surgeon. A 3D workflow based on an automated deep-learning approach was used to assess qualitative and quantitative morphometric changes in the position of the maxillary bone, as well as the distal and proximal segments of the mandible, by comparing preoperative and postoperative CBCT scans with the VSP.

## MATERIAL AND METHODS

A secondary analysis of preexisting data prospectively collected for clinical purposes was performed. The medical protocol and ethics followed the Declaration of Helsinki. The Institutional Review Board approved the study (HUM00224130 - reference for the University of Michigan, Ann Arbor, Mich). To use radiologic data for scientific analysis, a specific informed consent form was signed by all patients.

Deidentified CBCT scans (field of view of 17 × 20 cm; 400 μm; 110 kVp; 3.175 s; 59 mSV) from 17 nongrowing patients (9 females and 8 males), aged 18-32 years (mean age, 24.8 ± 3.5 years), with a Class III dentoskeletal malocclusion (A-point–Nasion-B-point angle <0°; unilateral or bilateral Angle Class III molar relationship), who underwent bimaxillary orthognathic surgery with traditional approach and preoperative VSP, were included. The VSP was conducted by an orthodontist in close communication with the surgeon using Dolphin Imaging software (Dolphin Imaging and Management Solutions, Chatsworth, Calif). After acquiring the presurgical CBCT scans, clinical records, and photographs, the orthodontist and maxillofacial surgeon discussed the treatment goals, including surgical, esthetic, and occlusal corrections. The orthodontist then conducted the VSP. This plan was presented to the surgeon for approval, with revisions made if necessary to align orthodontic and surgical objectives. Orthognathic surgery was performed using computer-aided design and manufacturing intraoperative splints for jaw repositioning. The CBCT scans were collected up to May 2023 and stored in the Dropbox folder at the laboratory-protected server at the University of Michigan, School of Dentistry. Patients with 1-jaw surgery, surgery-first approach, history of mandibular trauma, craniofacial syndromic malformations, systemic diseases, or osteometabolic disorders were excluded.

**Fig 1.**
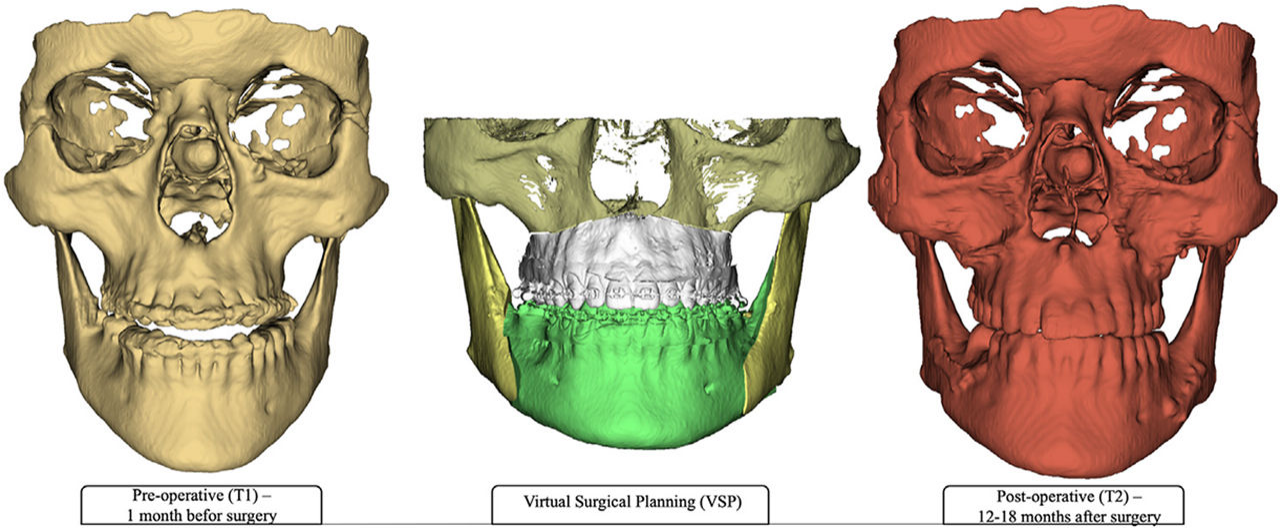
Three-dimensional reconstruction of T1 CBCT, VSP, and T2 CBCT. The automated segmentation of skeletal segments included the cranial base, maxilla, and mandible. Computer-assisted virtual surgical simulation by Dolphin Imaging software (Dolphin Imaging and Management Solutions, Chatsworth, Calif) involved both maxilla and mandible with 2-jaw surgery (LeFort I and bilateral sagittal split osteotomy).

Preoperative (T1) CBCT scans, performed 1 month before surgery, and postoperative (T2) CBCT scans taken at the midterm period, 12-18 months after surgery, were analyzed and compared with the VSP. The virtual simulation was performed by a trained orthodontist according to the objectives discussed with the maxillofacial surgeon to determine the jaw movements in all spatial directions (Fig 1). The resulting data were then exported as stereolithography files. Three-dimensional image analysis was conducted using the software 3D Slicer, using dedicated tools on the basis of recently validated deep-learning approaches.^26-28^ A standardized orientation of all T1 CBCTs was performed according to the Frankfurt plane and the midsagittal plane.^28,29^ Automated voxel-based registration of postoperative CBCTs on oriented T1 scans was performed for each patient, selecting the cranial base as the reference area of superimposition because it is not modified by the surgery.^28^ Automated bone segmentation allowed us to obtain the virtual models of each skeletal segment (cranial base, maxilla, and mandible). Quality control of the segmentations was performed using the ITK-SNAP software (version 3.8.0; http://www.itksnap.org).^26^ Automated landmarks identification was performed; landmark position was then checked by an experienced orthodontist (Table I).^27^

For the qualitative analysis, automated surface registration was performed to superimpose the skeletal models of VSP and T2. Shape analyses were conducted by subtracting the point-based models of VSP and T2, allowing for better visualization of changes that occurred in the 3 spatial axes (x, y, and z). Surgical outcomes were highlighted using semitransparent overlays and colormaps generated automatically by the software. By adjusting the surface distance values on the color bar, the interpretation of the distance maps enhanced the understanding of the magnitude of positional changes between the models, with excess or deficit movements being correlated with positive and negative numbers, respectively.

For the quantitative analysis, the dedicated tool “automatic quantification of 3D components (AQ3DC)” was used to calculate linear measurements in millimeters along the 3 coordinates: anteroposterior (x-axis), upper-lower (y-axis), and right-left (z-axis). Root mean square was used to calculate the 3D displacement. In addition, angular measurements in degrees were obtained for the 3 components: yaw, pitch, and roll. The focus of the analysis was on comparing T1-VSP and T1-T2 data. To assess the accuracy of mandibular and maxillary movements relative to the cranial base region of reference, displacements within 2 mm for linear measurements and within 4° for angular measurements were considered clinically acceptable criteria.^1^

Statistical analysis was performed using the R (version 4.3; R Core Team, Vienna, Austria). Intrarater reliability was preliminarily determined using the intraclass correlation coefficient for all measurements. A pilot study was conducted to determine the sample size needed for comparing 2 paired means. A total of 15 patients would be required, considering the following parameters: the difference in means (μ1 − μ2 = 1.47), the standard deviation (SD = 1.85), the significance level (α = 0.05), and the statistical power (β = 0.8). To evaluate the normality of each distribution, the Kolmogorov-Smirnov test was calculated. Descriptive statistics recorded absolute and relative frequencies for categorical data, mean and standard deviation for continuous quantitative variables with normal distribution and median and interquartile range for asymmetric distributions, and 95% confidence interval for a difference between 2 means. Bivariate analysis was performed using a 2-tailed Student *t* test for normal distributions and Wilcoxon and Mann-Whitney U tests for asymmetrical distributions. The level of significance was set at α = 0.05.

## RESULTS

**Fig 2.**
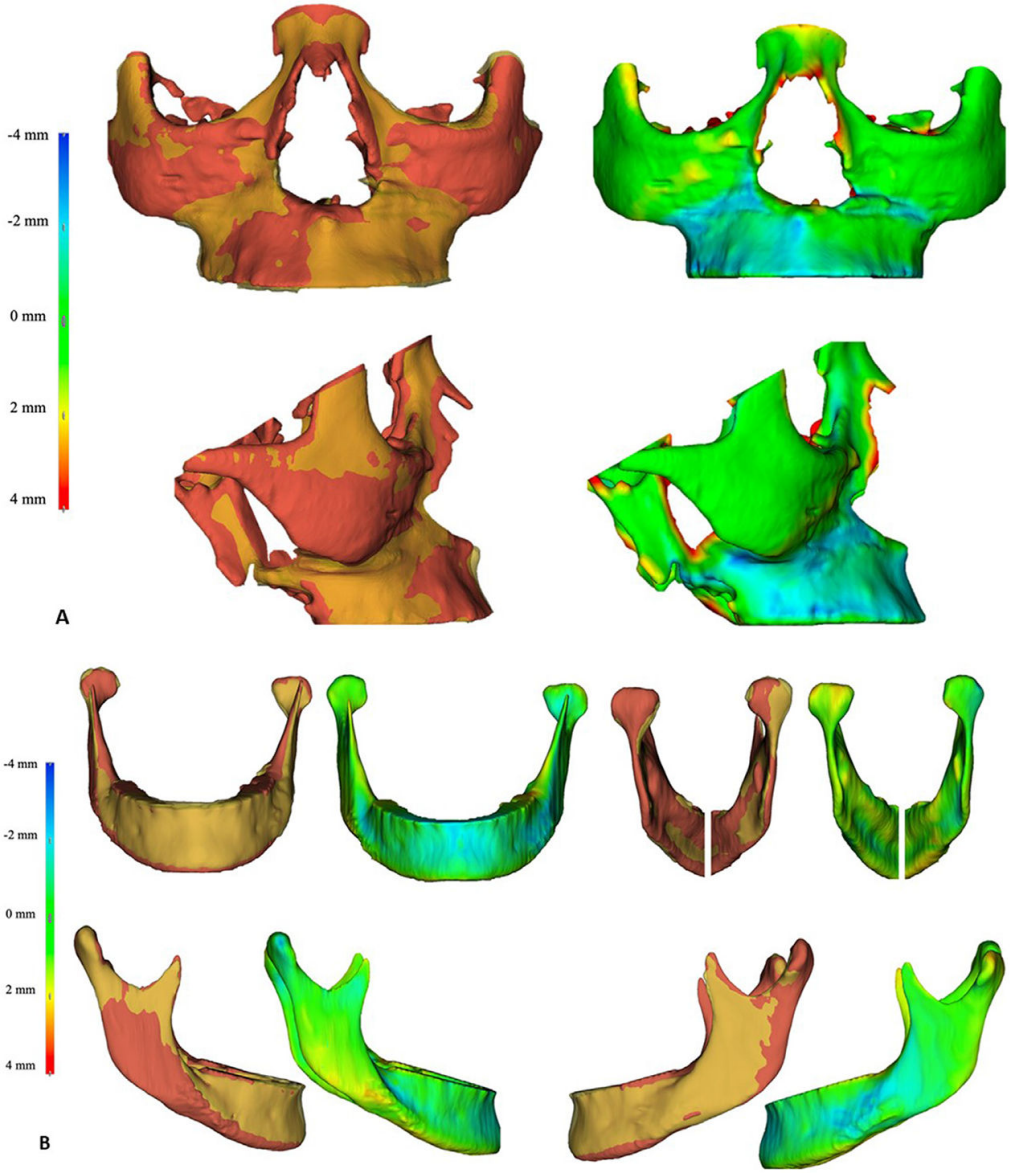
Qualitative analysis of a representative patient highlighting the differences between the virtual planning (*yellow*) and the postsurgical outcomes (*red*) at 12-18 months of follow-up. Semitransparent overlays of 3D models and corresponding color-coded maps are displayed using a color bar to show excess displacements (*red*) and deficits (*blue*). **A,** Frontal and lateral views of the maxillary bone reveal that the planned advancement was greater than the observed sagittal displacement. **B,** Frontal, posterior, and lateral views of the mandible show a slightly backward position of the condyles and a lateral displacement of the proximal segments.

Details of the study sample are presented in Table II. The mean duration of postsurgical follow-up was 15.40 ± 2.92 months. The intraclass correlation coefficient for all measurements exceeded 0.87, indicating high reliability. Figure 2 provides a qualitative analysis, displaying semitransparent overlays and color-coded maps of a representative patient to illustrate the findings. Tables III-V compare the results between T1-VSP (planned position) and T1-T2 (observed position), summarizing the linear and angular displacements, respectively. Table VI reports the cephalometric skeletal measurements at T1 (presurgical), T2 (postsurgical), and VSP (planned).

A statistically significant difference was observed in the postoperative advancement of the maxilla compared with the planned position, with a mean difference of 1.8 mm between T1-VSP and T1-T2 (*P* = 0.004). No significant differences were found in the maxillary pitch rotation between the planned and observed positions (slight nonsignificant anterior downward rotation, median values: T1-VSP, 3.57° (0.35-15.07); T1-T2, 3.10° [0.20-12.94]; *P* = 0.438). As the maxilla was advanced, no significant yaw (right-left) differences were found between the planned and observed positions (T1-VSP: 2.7 ± 1.9°; T1-T2: 3.3 ± 1.9°; *P* = 0.35).

A slight differential anteroposterior displacement of the distal segment at B-point was observed between T1-VSP (1.01 ± 3.66 mm) and T1-T2 (0.32 ± 4.17 mm); however, the difference was not statistically significant (*P* = 0.611). No significant differences in rotation of the mandibular plane were found between the planned (median value, 2.10 [0.03-9.30]) and midterm surgical outcomes (median value, 2.50 [0.11-8.74]) (*P* = 0.89). Both planned and midterm surgical results showed an increase in the mandibular gonial angle; however, the difference was not statistically significant (T1-VSP, 2.25°; T1-T2, 1.7°; *P* = 0.762). The yaw of the distal segment showed no significant difference between the planned and observed positions (T1-VSP, 1.53° ± 1.60°; T1-T2, 1.54° ± 1.50°; *P* = 0.986).

A slightly lower and posterior condylar position was observed, with no significant difference between T1-VSP and T1-T2 (mean sagittal difference, 0.29 mm; mean vertical difference, 0.09 mm; *P* >0.05). A slightly superior and anterior position of the anatomic gonial angle was detected, with no significant difference between T1-VSP and T1-T2 (mean sagittal difference, 0.16 mm; mean vertical difference, 1.02 mm; *P* >0.05). Intergonial distance showed a slight increase without statistical significance (T1-T2: 0.64 mm; T1-VSP: 0.04 mm; *P* = 0.78). Small mandibular right and left ramus lateral roll rotations within 1.5° were observed for both T1-VSP and T1-T2 (*P* >0.05).

## DISCUSSION

This study aimed to investigate the midterm surgical outcomes for the correction of Class III malocclusion using a VSP tool readily available in orthodontic practices, namely Dolphin 3D Imaging. This study offered several unique contributions to comprehensively evaluate midterm outcomes of orthognathic surgery. First, our findings demonstrated the remarkable predictability of jaw positions in the midterm period when using VSP. Second, this study highlighted the feasibility of orthodontists performing VSP in collaboration with the surgeon, emphasizing the importance of their active involvement. Third, the use of automated image analysis tools to quantitatively assess midterm surgical outcomes provided objective measurements of the position of the maxillary bone, as well as the distal and proximal segments of the mandible. By examining these aspects, this study aimed to provide valuable insights into the efficacy and potential advantages of using Dolphin 3D Imaging for orthodontic-surgical planning, shedding light on its potential impact on long-term stability and patient outcomes.

Although previous studies have frequently reported less stability of the mandible as a skeletal segment after orthognathic surgery, the findings in this midterm follow-up cohort indicated favorable predictability of the mandible after surgery.^18^ Specifically, the distal segment of the mandible showed an average 3D displacement exceeding 6 mm, which aligned with the anticipated position on the basis of the VSP. In a previous study using a similar methodology but without VSP, De Paula et al^18^ identified the chin and inferior border of the mandible as regions demonstrating the highest degree of postsurgical adaptation 1-year postsurgery. These regions exhibited adaptive changes >2 mm or ≤2 mm in most patients. The more favorable mandibular position in this study could likely be attributed to the implementation of VSP, which effectively predicts skeletal displacements in all directions. The largest difference between planned and actual outcomes was noted during sagittal analysis, with a minimal differential displacement of 0.7 mm. It is worth noting that although this difference fell within the 1-mm range, minor fluctuations might also be associated with vertical changes, which exhibited the most significant variation (actual midterm 2.6 mm vertical displacement at B-point and planned 2.4 mm) in this cohort. As reported in the literature, predicting the center of rotation of the jaws during VSP can be challenging, and if an autorotation occurs, it can impact the actual outcome.^18^ There is a mandibular adaptation after surgery that involves clock-wise or counterclockwise rotation, further altering the sagittal and vertical position of the mandible.^18^ In this cohort, a similar counterclockwise mandibular rotation, 0.4° difference, was observed at the midterm follow-up compared with the planned outcomes, which could also explain the minimal discrepancy in the maxillomandibular relationship. The anteroposterior component of the distance between the A-point and B-point showed lower values for T1-T2 than for T1-VSP, consistent with the observations made by Badiali et al^30^ in their Wits analysis.

The analysis of the mandibular proximal segments revealed a remarkable level of predictability in preserving their position. It is well-documented in the literature that the most substantial postoperative changes generally take place within the initial 6-8 weeks after surgery, as the restoration of function and movement between proximal and distal segments occurs.^31-33^ In this cohort, all mean proximal segment displacements were found to be <1 mm after an average follow-up of 15 months. These results indicated smaller displacements compared with the reported movements of the mandibular rami documented by De Paula et al,^18^ which exceeded 2 mm 1-year postsurgery.^18^ Condylar movements during surgery may be inevitable; however, the proximal segments can adapt to their physiological position, facilitating functional recovery and preventing the exacerbation of temporomandibular joint symptoms.^32,34,35^ In this study, both the T1-VSP and T1-T2 models exhibited an inferior and posterior small movement of the condyles, with no significant differences observed in the intercondylar distance. These findings aligned with the conclusions of Kim et al,^36^ who reported a posterior displacement of the condyles at the 12-month follow-up compared with the presurgical conditions. Furthermore, the literature has documented minor changes in condylar position resulting from slight angular movements 1-year postsurgery.^18,32^ Similar to the findings of these authors, this study revealed a lateral angulation of the mandibular ramus and a lateral, anterior, and superior position of the anatomic gonial angle. Importantly, no significant differences were observed between the planned and actual outcomes. The lateral angulation of mandibular rami may be attributed to the intricate interaction between the proximal and distal mandibular segments after bilateral sagittal split osteotomy, which poses challenges in achieving precise alignment.^32,36,37^ It is worth noting that despite the use of virtual surgical prediction, accurately anticipating intraoperative bone discrepancies can be complex. Even with minimal displacements, variations between software-simulated osteotomies and the actual surgical procedure may arise.^37^

When assessing the stability of the maxillary segment, it is essential to consider the contemporary trend of advancing the maxillomandibular complex in alignment with esthetic parameters. This approach is accompanied by an increasing preference for the use of segmental osteotomies.^38^ In this midterm surgical follow-up, maxillary advancement showed an average value slightly exceeding 3 mm, which is consistent with the results reported in the literature at the same time point.^18,39^ If compared with the VSP, a reduced advancement of the maxilla was observed, with a mean difference of 1.8 mm. This finding is not uncommon in orthognathic surgery, as previous studies have reported similar discrepancies.^18,30,38-40^ At the end of orthognathic and orthodontic treatment, Badiali et al^30^ observed a sagittal maxillary deficiency of 1 mm when comparing the expected outcomes with the actual results. Although De Paula et al^18^ conducted their assessment without the use of virtual simulation, they reported that at the time of splint removal, maxillary advancement consistently exceeded 4 mm. However, 1-year postsurgery, they observed postsurgical adaptation with changes ranging 2-4 mm in 52.2% of the patients. Kim et al^39^ compared virtual planning with postsurgical CBCT scans at 3 different time points (3 days, 4 months, and 1 year) after orthognathic surgery. They utilized customized cutting guides and fixation plates in their study. The authors also observed a posterior repositioning of the A-point over time, with a difference of approximately 1 mm. In this study, the maxillary repositioning was guided intraoperatively by the mandible without the use of customized plates or navigation systems. For the assessment of the discrepancies between the planned and actual maxillary advancement, it would be helpful to consider the amount of planned advancement.^38^ In this patient cohort, the projected maxillary advancement ranged from 2 to 10 mm, with an average of 4.94 mm. Notably, within the group of 6 patients who displayed a maxillary discrepancy exceeding 2 mm between T1-VSP and T1-T2, 4 of which had planned advancements that exceeded 5 mm. The interpretation of less maxillary advancement should also consider aspects under the surgeon’s control.^30,38^ During intraoperative maneuvers, there is a tendency for posterior condylar movements because of the supine position of the patient under general anesthesia. If a correct centric relation is not achieved during maxillary repositioning, particularly by applying a forward force vector at the mandibular angles, it could result in posterior displacement of the maxilla, interfering with the virtual and actual outcomes.^38^ Because the maxillary position was evaluated on the basis of the 3D coordinates of the A-point, postoperative evaluation should also consider all procedures affecting the maxillary dentoalveolar process. The observed postsurgical modifications could also be a consequence of orthodontic movements after surgery, particularly related to the maxillary incisors or intraoperative esthetic procedures performed to enhance the outcome of orthognathic surgery. Previous studies indicated that it is not an uncommon surgical procedure to reshape the anterior nasal spine and the anterior concavity of the premaxilla, which may result in a more posterior displacement of the A-point.^30,32^ Considering the multiple factors involved in maxillary repositioning, the virtual planned movement of the maxilla could benefit from a slight sagittal overcorrection, particularly if the esthetic goals of orthognathic surgery include better dental exposure during smiling and improved support for soft tissues.

The involvement of an experienced orthodontist using the Dolphin Imaging VSP module in this study provided valuable insights and feedback to the surgeon, benefiting from the orthodontist’s expertise in postsurgical orthodontic treatment and contributing to optimizing the overall planning of skeletal modifications. Although the pursuit of esthetic goals remains paramount, a robust plan should also consider potential surgical intricacies that could hinder optimal jaw movements. In addition, the prediction of postsurgical occlusal relationships should be considered to facilitate subsequent orthodontic finalization. Evaluating the final occlusal relationships should aid in planning future tooth movements concerning both hard and soft-tissue support. Both the active involvement of the surgeon and the participation of the orthodontist are pivotal elements, primarily for the virtual prediction of the final occlusal stability, a key factor influencing postsurgical skeletal and dentoalveolar stability. In contemporary practice, computer-assisted technology has become ubiquitous, and the accessibility of in-house 3D printers in both orthodontics and oral-maxillofacial surgery practices has facilitated the integration of virtual surgical simulation software into orthodontic-surgical work-flows. Consequently, it is imperative for all orthodontists to acquaint themselves with VSP software to augment their communication with surgeons. Moreover, the direct involvement of an orthodontist in the VSP process ensures a comprehensive and customized approach, maximizing the potential for successful long-term outcomes and patient satisfaction.

The 3D image analysis used an automated voxel-based digital protocol and accurate open-source software based on convolutional neural networks, allowing a qualitative and quantitative interpretation of postsurgical changes. The use of artificial intelligence-based tools ensured reliable assessment for postoperative evaluation by automating superimposition and minimizing operator dependency. A potential study limitation arises from the absence of an immediate postoperative CBCT scan, driven by ethical considerations in minimizing participants’ radiation exposure. As a result, the ability to definitively distinguish among relapse, postsurgical adaptation, and dynamic instability is somewhat constrained. However, when comparing the findings of this study with the existing literature, the significant impact of VSP becomes evident. In the midterm surgical follow-up of this study, the use of VSP has resulted in surgical accuracy and precise prediction of skeletal movements of the jaws and facilitated 3D evaluation of patients.

## CONCLUSIONS

At the 12- to 18-month follow-up, minimal postsurgical discrepancies in the positioning of the maxilla and mandible compared with the planned movements were observed. A reduced maxillary advancement was observed, suggesting that a slight sagittal overcorrection in the virtual planned movement of the maxilla might be beneficial. Establishing a direct collaboration between surgeons and orthodontists in the development of the virtual surgical simulation is crucial to enhancing long-term skeletal stability and achieving improved postoperative outcomes.

## Figures and Tables

**Table I.** Description of cephalometric landmarks for skeletal analysis

Landmarks	Abbreviation	Definition	Abbreviation of bilateral landmarks
Maxilla			
Orbitalis	Or	The most inferior point on the lower portion of the orbit contour	Right side: ROr; left side: LOr
Medial zygomatic point	MZyg	The deepest portion of the maxillary zygomatic process curvature	Right side: RMZyg; left side: LMZyg
A-point	A	The most posterior point of the anterior concavity of the maxilla	–
Mandible			
B-point	B	The most posterior point of the anterior concavity of the mental symphysis	–
Pogonion	Pog	The most anterior point of the mentonian symphysis	–
Menton	Me	The most inferior point of the mentonian symphysis	–
Condylion	Co	The most superior point of the condyle contour	Right side: RCo; left side: LCo
Medial condylar pole	MCo	The most medial and central point of the condyle	Right side: RMCo; left side: LMCo
Lateral condylar pole	LCo	The most lateral and central point of the condyle	Right side: RLCo; left side: LLCo
Gonion	Go	The most inferior and posterior point of the mandibular angle	Right side: RGo; left side: LGo

**Table II.** Descriptive data of the study population

Demographic variables	Study sample
Patients	17
Sex	
Female	9 (53%)
Age (y)	24.8 ± 3.5
Sagittal skeletal diagnosis	
Class III malocclusion	17 (100%)
Skeletal asymmetry	9 (53%)
Mean follow-up (mo)	15.40 ± 2.92

Note. Values are shown as mean ± standard deviation or number (percentage).

**Table III.** Linear displacements of skeletal landmarks between T1-VSP and T1-T2 models

*Linear displacement (mm)* *Landmarks*	*Group*		P *value*
	*T1-VSP*	*T1-T2*	
Maxilla			
A-point			
AP	4.94 ± 1.88	3.10 ± 1.60	0.004*
SI	0.53 (−2.50 to 5.96)	0.20 (−1.97 to 6.25)	0.918
3D	5.54 ± 1.85	4.01 ± 1.86	0.020*
MidZyg			
RL	−0.20 ± 1.16	−0.11 ± 1.10	0.816
AP	0.00 (−3.00 to 4.95)	0.44 (−1.08 to 2.29)	0.581
SI	−0.44 (−2.50 to 2.11)	−0.07 (−2.50 to 1.69)	0.215
3D	1.60 (0.40-5.55)	1.46 (0.31-2.85)	0.326
MidOr			
RL	−0.19 ± 0.89	−0.25 ± 0.83	0.842
AP	0.04 ± 1.14	0.02 ± 0.71	0.975
SI	0.13 ± 0.64	−0.11 ± 0.47	0.219
3D	1.43 ± 0.61	1.11 ± 0.43	0.084
Mandible			
B-point			
RL	0.26 ± 2.58	0.91 ± 3.11	0.510
AP	1.01 ± 3.66	0.32 ± 4.17	0.611
SI	2.39 (−2.45 to 14.96)	2.58 (−1.14 to 11.9)	0.389
3D	5.15 (2.52-15.27)	6.65 (2.56-12.45)	0.143
Pog			
RL	−0.01 ± 2.88	0.99 ± 3.10	0.335
AP	1.98 ± 4.77	1.89 ± 4.82	0.955
SI	1.82 (−1.89 to 10.85)	1.65 (−0.59 to 10.26)	0.679
3D	6.52 ± 3.49	6.75 ± 3.10	0.843
Me			
RL	−0.15 ± 3.12	0.84 ± 3.31	0.376
AP	1.84 ± 4.70	2.29 ± 5.02	0.789
SI	1.38 (−1.21 to 12.21)	1.4 (−0.91 to 12.36)	0.973
3D	6.35 (1.46-14.94)	7.47 (1.64-14.38)	0.370
MidCo			
RL	−0.20 ± 0.88	−0.25 ± 0.58	0.845
AP	−0.39 (−3.35 to 0.84)	−0.10 (−3.35 to 1.08)	0.153
SI	−0.19 (−5.07 to 2.00)	−0.10 (−5.07 to 0.58)	0.679
3D	1.58 (0.48-6.18)	0.97 (0.12-6.18)	0.006*
MidLCo			
RL	−0.06 ± 0.74	−0.05 ± 0.55	0.973
AP	−0.48 (−4.25 to 0.71)	0.28 (−4.25 to 1.51)	0.020*
SI	0.04 (−2.07 to 3.92)	0.01 (−2.07 to 1.19)	0.705
3D	1.57 (0.38-4.81)	0.99 (0.28-4.81)	0.174
MidMCo			
RL	0.23 (−1.57 to 1.07)	−0.02 (−1.05 to 0.33)	0.270
AP	0.14 (−1.59 to 3.77)	−0.38 (−1.59 to 1.22)	0.153
SI	−0.52 ± 1.65	−0.76 ± 1.27	0.643
3D	1.59 (0.33-4.50)	0.91 (0.21-4.50)	0.079
MidGo			
RL	0.19 ± 1.19	0.43 ± 1.68	0.636
AP	0.39 ± 1.75	0.55 ± 2.01	0.808
SI	−0.57 ± 1.33	0.45 ± 1.69	0.060
3D	2.39 ± 0.87	3.04 ± 0.77	0.026*

Note. Values are shown as mean ± standard deviation or mean (95% confidence interval).

*RL*, right-left (a positive value means right displacement); *AP*, anteroposterior (a positive value means anterior displacement); *SI*, superior-inferior (a positive value means superior displacement); *3D*, root mean square.

* Statistically significant result.

**Table IV.** Maxillary repositioning discrepancy distribution

Anteroposterior discrepancy	n (%)
>2 mm	6 (35.3)
≤2 mm	11 (64.7)
1-2 mm	5 (45.5)
<1 mm	6 (54.5)

**Table V.** Angular displacements between T1-VSP and T1-T2 models

*Angular* *displacement (°)* Rotational movement	*Group*		P value
	T1-VSP	T1-T2	
Maxilla			
Maxilla (MidZyg-A)			
Yaw	2.64 ± 1.89	3.28 ± 2.00	0.350
Pitch	3.57 (0.35-15.07)	3.57 (0.35-15.07)	0.438
Mandible			
Mandible (MidGoMe)			
Yaw	1.53 ± 1.57	1.54 ± 1.48	0.986
Pitch	2.10 (0.03-9.30)	2.50 (0.11-8.74)	0.890
Left ramus (LCo-LGo)			
Roll	1.23 ± 0.77	1.53 ± 1.22	0.392
Right ramus (RCo-RGo)			
Roll	1.32 (0.34-4.55)	1.23 (0.00-4.98)	0.931

Note. Values are shown as mean6standard deviation or mean (95% confidence interval).

**Table VI.** Comparative assessment of 3D cephalometric skeletal measurements

*Skeletal Measurements*	*Group*				P *value*
*Distance*	*Component*	*T1*	*VSP*	*T2*	
Maxilla					
Maxillary projection (MidZyg-A)	AP (mm)	24.38 ± 2.17	28.89 ± 2.47	27.06 ± 2.08	0.026*
Mandible					
Mandibular gonial angle (CoGoMe)	Pitch (°)	54.58 ± 5.38	56.83 ± 5.42	56.28 ± 5.07	0.762
		M: 54.75 ± 5.12	M: 57.56 ± 4.54	M: 57.18 ± 2.66	
		F: 54.42 ± 5.9	F: 56.19 ± 6.3	F: 55.49 ± 6.61	
Mandibular projection (MidGo-B)	AP (mm)	68.79 ± 5.51	69.41 ± 5.56	68.56 ± 6.19	0.677
Mandibular projection (MidGo-Pog)	AP (mm)	70.93 ± 5.44	72.52 ± 6.17	72.27 ± 5.98	0.904
Mandibular body length (MidGo-Me)	AP (mm)	65.26 ± 5.03	66.70 ± 5.80	67.00 ± 5.91	0.885
Mandibular right ramus height (RCo-RGo)	SI (mm)	60.66 ± 5.02	60.10 ± 4.78	59.65 ± 4.88	0.789
		M: 64.13 ± 2.96	M: 62.91 ± 3.01	M: 62.42 ± 3.06	
		F: 57.56 ± 4.46	F: 57.60 ± 4.79	F: 57.19 ± 5.0	
Mandibular left ramus height (LCo-LGo)	SI (mm)	58.06 ± 5.89	58.79 ± 5.61	57.51 ± 6.12	0.528
		M: 61.48 ± 3.35	M: 61.73 ± 2.03	M: 61.16 ± 3.51	
		F: 55.01 ± 6.12	F: 56.18 ± 6.55	F: 54.27 ± 6.25	
Mandibular left body length (LGo-Me)	AP (mm)	66.13 ± 4.69	67.59 ± 5.38	68.18 ± 5.23	0.748
Mandibular right body length (RGo-Me)	AP (mm)	64.39 ± 5.76	65.82 ± 6.68	65.82 ± 7.20	0.999
Intercondylar distance (RCo-LCo)	RL (mm)	98.46 ± 5.22	98.60 ± 5.14	97.84 ± 5.40	0.677
		M: 99.95 ± 4.45	M: 99.71 ± 4.0	M: 99.7 ± 4.04	
		F: 97.14 ± 5.75	F: 97.62 ± 6.05	F: 96.19 ± 6.12	
Intergonial distance (RGo-LGo)	RL (mm)	88.4 ± 5.79	88.44 ± 6.33	89.04 ± 6.08	0.78
		M: 92.86 ± 5.04	M: 93.50 ± 5.32	M: 93.34 ± 5.14	
		F: 84.45 ± 2.73	F: 83.95 ± 2.65	F: 85.22 ± 3.97	
Maxillomandibular relationship					
A-B distance	AP (mm)	−3.60 ± 4.28	0.33 ± 3.07	−0.82 ± 3.5	0.316

Note. Values are shown as mean ± standard deviation or mean.

*RL*, right-left; AP, anteroposterior; *SI*, superior-inferior; *M*, male; *F*, female.

* Statistically significant result.
